# Neuroprotective Activities of *Boophone haemanthoides* (Amaryllidaceae) Extract and Its Chemical Constituents

**DOI:** 10.3390/molecules25225376

**Published:** 2020-11-17

**Authors:** Abobaker S. Ibrakaw, Sylvester I. Omoruyi, Okobi E. Ekpo, Ahmed A. Hussein

**Affiliations:** 1 Department of Biodiversity and Conservation Biology, University of the Western Cape, Cape Town, Robert Sobukwe Road, Bellville 7535, South Africa; 3686844@myuwc.ac.za; 2 Department of Chemistry, Cape Peninsula University of Technology, Symphony Road, Bellville 7535, South Africa; omoruyis@cput.ac.za; 3 Department of Medical Biosciences, University of the Western Cape, Cape Town, Robert Sobukwe Road, Bellville 7535, South Africa; oekpo@uwc.ac.za

**Keywords:** Amaryllidaceae, *Boophone haemanthoides*, alkaloids, terpenoids, Parkinson’s disease, neuroprotection, apoptosis

## Abstract

Parkinson’s disease (PD) is a neurodegenerative condition that progresses as age increases, and some of its major symptoms include tremor and postural and movement-related difficulties. To date, the treatment of PD remains a challenge because available drugs only treat the symptoms of the disease or possess serious side effects. In light of this, new treatment options are needed; hence, this study investigates the neuroprotective effects of an organic *Boophone haemanthoides* extract (BHE) and its bioactive compounds using an in vitro model of PD involving the toxin 1-methyl-4-phenylpyridinium (MPP^+^) and SH-SY5Y neuroblastoma cells. A total of seven compounds were isolated from BHE, viz distichamine (**1**), 1α,3α-diacetylnerbowdine (**2**), hippadine (**3**), stigmast-4-ene-3,6-dione (**4**), cholest-4-en-3-one (**5**), tyrosol (**6**), and 3-hydroxy-1-(4′-hydroxyphenyl)-1-propanone (**7**). Six compounds (**1**, **2**, **4**, **5**, **6** and **7**) were investigated, and five showed neuroprotection alongside the BHE. This study gives insight into the bioactivity of the non-alkaloidal constituents of Amaryllidaceae, since the isolated compounds and the BHE showed improved cell viability, increased ATP generation in the cells as well as inhibition of MPP^+^-induced apoptosis. Together, these findings support the claim that the Amaryllidaceae plant family could be a potential reserve of bioactive compounds for the discovery of neuroprotective agents.

## 1. Introduction

Parkinson’s disease (PD) is a neurodegenerative disease that worsens with increasing age and affects about 10 million people worldwide. The initial manifestations of the disease occur at approximately 60 years of age, with females being less susceptible to the disease than males [1,2]. Although the incidence of PD has been strongly linked to age, a cross sectional study showed that approximately 30% of PD patients are younger than 65 years of age at the time of diagnosis [3]. The major symptoms of PD include tremor and postural and movement-related disorders [2]. These PD-related symptoms arise from the profound and selective loss of dopaminergic neurons in the substantia nigra pars compacta of the midbrain and the formation of Lewy bodies in the cytoplasm of neuronal cells.

Although the etiology of PD is not fully understood, studies have shown that the loss of dopaminergic neurons can be associated with a number of factors, key among which are oxidative stress and mitochondrial dysfunction [4,5]. Biochemically, the earliest signs of PD involve the impairment of the mitochondrial electron chain impairment, alteration of mitochondrial dynamics, and an imbalance in calcium and iron homeostasis [6]. Following these changes, there is increased reactive oxygen species (ROS) generation in the mitochondria of neuronal cells, leading to a defect in the functioning of mitochondrial complex I, which is believed to be a major contributor to dopaminergic neuronal cell degeneration in PD [7,8]. In addition, the synthesis of adenosine triphosphate (ATP) is negatively affected, and the reduction of cellular ATP in turn drives dopaminergic neurons into programmed cell death (PCD) [9].

To understand the progression of PD in laboratory studies, the non-toxic chemical 1,2,3,6-methyl-phenyl-tetrahydropyridine (MPTP) is often used to model the disease in vivo [10,11]. Upon crossing the blood–brain barrier (BBB), MPTP is converted by the enzyme mono amine oxidase B (MAO-B) in astrocytes into the toxic metabolite 1-methyl-4-phenylpyridinium (MPP^+^), which leads to mitochondrial dysfunction [12]. MPP^+^ is known to cause the mitochondrial permeability transition pore to open, which in turn leads to a change in the mitochondrial membrane potential and increased ROS accumulation in the cells, which alters ATP levels and eventually induces apoptosis [6].

Although there is no specific cure for PD, levodopa, which is a dopamine-replacement therapy, is currently in use for the treatment of PD symptoms. However, the prolonged use of levodopa has been shown to be associated with some side effects, including the enhancement of oxidative stress and the acceleration of degeneration of residual dopaminergic neurons in PD patients using this medication [9], thus necessitating the search for alternative treatment options. A number of in vitro and in vivo studies have shown that herbal medicines, phytochemicals, and other plant-derived bioactive compounds and dietary supplements could ameliorate the effects of PD [13,14,15].

*Boophone haemanthoides* is a deciduous, winter-growing bulb plant that survives in almost all weather conditions, including the moist winter season, as well as in hot and dry summer temperatures. It belongs to the Amaryllidaceae plant family and is endemic to the winter and rainfall regions of South Africa and Namibia [16]. This plant family, which comprises over 800 species and 80 genera, is well distributed in the tropical regions of the world and found in abundance in the Southern Africa region of Africa [17,18]. Plants in this family are well known for their alkaloids, and so far more than 630 alkaloids have been isolated from these plants, many of which are known to possess a number of biological activities including antibacterial, anti-cancer, and neuroprotective activities [19,20,21].

*B. haemanthoides* was reported to be used traditionally by the Khoi-San tribe in the Northern Cape Province of South Africa for the treatment of asthma and for relieving knee pain [22]. Furthermore, a number of bioactive Amaryllidaceae alkaloids have been isolated from *Boophone disticha*, another member of the genus *Boophone*, including distichamine, buphanidrine, buphanisine, crinine, and distichaminol [23,24,25]. In one study, *B. disticha* was reported to show neuroprotective activities in 6-hydroxydopamine (6-OHDA)-induced SH-SY5Y toxicity by inhibiting ATP degeneration [26], while distichamine, buphanidrine, and buphanisine have also been reported to show a strong affinity for the neurotransmitter serotonin [20]. Importantly, the approval of galanthamine, an Amaryllidaceae alkaloid, for the treatment of Alzheimer’s disease by the US food and drug administration (FDA) has made alkaloids from this plant family potential sources of novel neuroprotective agents [27]. Thus, the present study investigates the neuroprotective activities of *B. haemanthoides* and its isolated compounds on MPP^+^-induced neuronal toxicity in SH-SY5Y neuroblastoma cells.

## 2. Results

### 2.1. Isolation and Identification of the Chemical Constituents

**Figure 1:**
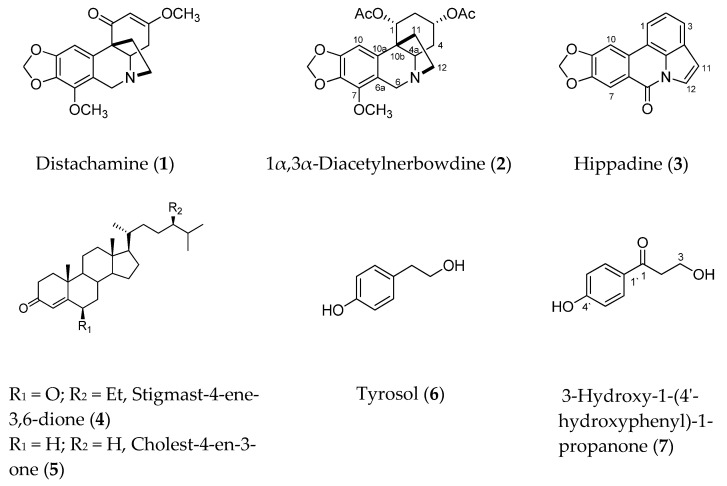
Chemical structures of compounds **1**–**7** isolated from *B. haementhoids*.

*B. haementhoids* collected in South Africa were previously investigated, and eight alkaloids, including distichamine (**1**), were described [25]. Recently, triterpenes and other minor constituents including compounds **4**–**7** were described in our previous publication [28]. In this study we report on the isolation and identification of three additional known alkaloids, *viz* distichamine (**1**), 1α,3α-diacetylnerbowdine (**2**), and hippadine (**3**) (Figure 1), as well as the neuroprotection potential of compounds **1**, **2**, **4**–**7** against MPP^+^-induced toxicity in an in vitro PD model.

Compound **3** (hippadine) was isolated as minor constituent and identified according to its ^1^H-NMR and GC–MS analysis data, which was compared with available data in literature [29].

Compound **2** is described here as a natural product for the first time. It showed a typical positive reaction of alkaloids with Dragendorff reagent on TLC. The GC–MS analysis showed a single peak at Rt 36.405 min, with *m*/*z* 403.4, corresponding to molecular formula C_21_H_25_NO_7_. ^1^H-NMR showed that an aromatic signal appeared at *δ*_H_ 6.11 (*s*, H_10_); two signals of methylenedioxy protons at 5.83, 5.79 *d* each (*J* = 1.3 Hz) (OCH_2_O); two proton signals of two methines appeared at 5.71 *br t* (*J* = 2.6 Hz) and 5.15 *br quint* (*J* = 2.6 Hz) of H_1_ and H_3_, respectively; two signals of a methylene group at 4.13, 3.76 *d* each (*J* = 17.3 Hz) and belong to C_6_; a proton signal at 3.48 *dd* (*J* = 5.5, 12.2 Hz); two acetoxy groups at 1.97 and 1.90; a methoxy at 3.95, in addition to signals of four methylene groups (C_11_, C_12_, C_2_, and C_4_) (Table 1). ^13^C-NMR showed 21 carbon signals classified using DEPT-135 and HSQC into a methylenedioxy carbon (*δ*_C_ 100.5), a quaternary carbon (*δ*_C_ 46.8, C_10b_), six aromatic carbons including a methine (*δ*_C_ 117.2, 140.4, 133.2, 148.4, 97.1), a methoxy (*δ*_C_ 59.1), six methylene (*δ*_C_ 28.6, 30.5, 57.5, 38.2, 51.0, 100.5 of C_2_, C_4_, C_6_, C_11_, C_12_, and OCH_2_O, respectively), and two oxygenated methines (*δ*_C_ 68.3 and 68.0), and two acetates (21.2/170.5, 21.3/170.1).

**Figure 2:**
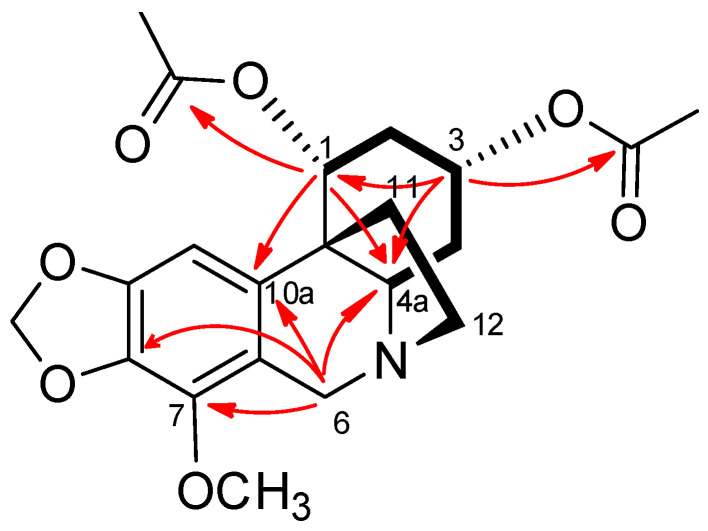
Important COSY (▬) and HMBC (H→C) correlations of compound **2**.

Comparison of the given NMR data with literature indicated a crinane alkaloid with two acetoxy groups and a methoxy group, as shown in Figure 1. Other 2D NMR experiments (COSY, HMBC, and NOESY) confirmed the structure and the positions of the two acetates and the methoxy groups. The COSY spectra (Figure 2) showed correlations (coupling) of the methylene protons at C_2_ with the methine protons at C_1_ and C_3_, while the H_1_ only coupled with H_2_ protons; in addition, the methylene protons at C_4_ had correlations with the methines protons at C_3_ and C_4a_, which indicated the positions of the two acetate groups at C_1_ and C_3_. The positions were further confirmed by HMBC spectra (Figure 2), which showed correlations between H_2_ and carbons C_4_, C_10b_, C_1_, C_3_; H_1_ and C_2_, C_10b_, C_3_, C_4a_, C_10a_, and CO; H_3_ and C_2_, C_4_, C_4a_, C_1_, and CO; and H_4a_ and C_4_, C_11_, C_12_, C_6_, and C_10a_. On the other hand, the methoxy group was allocated at C_7_ from the HMBC correlations of the methoxy protons with C_7_; H_10_ and C_10a_, C_6a_, C_10b_, C_7_, and C_9_; and H_6_ and C_12_, C_4a_, C_6a_, C_10a_, and C_7_. After careful literature review, we proposed the structure given in Figure 1.

**Figure 3:**
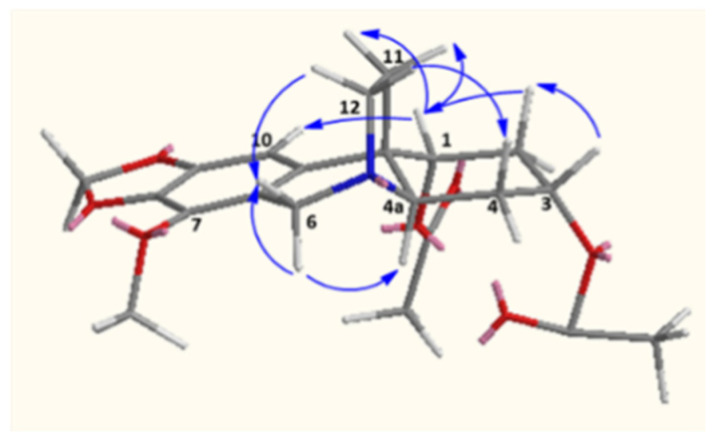
Important NOESY correlations of compound **2**.

The beta orientation of C_11_ and C_12_ were proposed tentatively due the fact that the compound has the same optical rotation with nerbowdine, which was isolated from the same species and was the precursor of **2**. The alpha orientation of the acetate groups at position C_1_ and C_3_ was partially proved by the weak coupling of the equatorial protons at C_1_ and C_3_. On the other hand, NOESY spectra showed correlations between H_1β_/H_10_, H_2α_, H_2β_ H_11exo_, and H_11endo_; while H_3β_ showed correlation with H_2α_, H_2β_, H_4α_, and H_4β_, and these indicate the equatorial protons (at C_1_ and C_3_) are in β positions (Figure 3).

This is the first report on the isolation of the diacetate from a natural source. However, the same compound **2** was described in the literature as the diacetate synthetic derivative of nerbowdine (also known as haemanthine, hemanthine, and buphantine) [30].

Distichamine has been previously reported from *B. haementhoides* [25], *B. distichia* [20], and *Amaryllis belladonna* [31]. The isolation of mixed alkaloidal skeletons such as crinine (compounds **1** and **2**) and lycorine (**3**) types is a common feature of the alkaloid biosynthesis from all members of the amarylloideae subfamily (Amaryllidaceae), which reflects the ability of the dynamic enzymatic systems of these subfamily members to biosynthesize different skeletons from the 4′-*O*-methylnorbelladine [32].

### 2.2. Dose Response of BHE and Isolated Compounds

**Figure 4:**
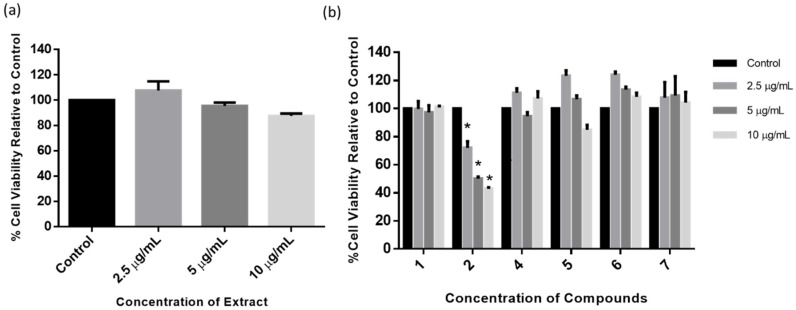
Dose–response of BHE and compounds. MTT assay cytotoxicity on SH-SY5Y cells treated with increasing concentrations (2.5, 5, and 10 µg/mL) of (**a**) BHE and (**b**) compounds for 24 h, and each bar represents mean cell viability expressed as percentage of control; * indicates significance at *p* < 0.05.

In order to ascertain the optimum concentrations of BHE and isolated compounds to be used for neuroprotection studies, an MTT cytotoxicity assay was performed in SH-SY5Y cells treated with 2.5, 5, and 10 µg/mL of either extracts or individual compounds. Figure 4a shows that BHE had no impact on SH-SY5Y cell viability, and the 2.5 µg/mL concentration increased cell viability the most (107.65%). Furthermore, all compounds showed either increased or had no significant effect in cell viability at all concentrations tested, except for compound **2**, which showed a significant reduction in cell viability at all treatment concentrations (Figure 4b). While it might be possible that lower concentrations of compound **2** may have yielded a better outcome, it was, however, not tested and as such we cannot be certain. Taken together, these results indicate that BHE and isolated compounds showed no cytotoxicity in SH-SY5Y cells at the tested concentrations and the 2.5 µg/mL concentration was chosen for further neuroprotection studies.

### 2.3. Dose Response of MPP^+^ in SH-SY5Y

**Figure 5:**
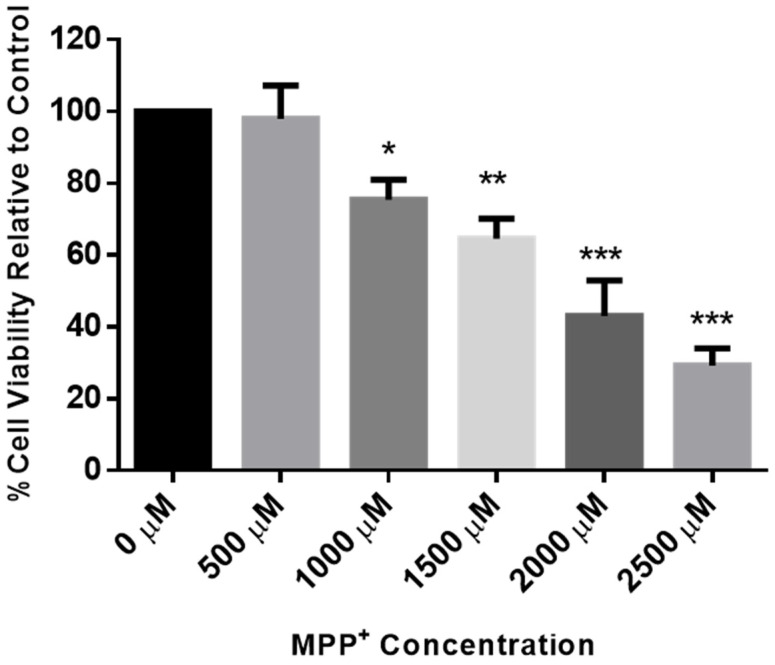
Dose–response of MPP^+^ in SH-SY5Y cells. SH-SY5Y cells were treated with MPP^+^ (500 µM–2500 µM), and after 24 h cell viability was assessed using MTT assays. Each bar represents mean percentage cell viability, and significance of difference is indicated with * (*p* < 0.05), ** (*p* < 0.01), and *** (*p* < 0.001).

To confirm the concentration of MPP^+^ to be used for evaluating neuronal toxicity in the SH-SY5Y cells, the MTT cytotoxicity assay was again performed following exposure of the cells to a range of 500 to 2500 µM of MPP^+^ for 24 h. Figure 5 shows a concentration-dependent decrease in the viability of the cells, and the 2000 µM concentration, which was found to reduce cell viability to about 43% when compared to the control, was chosen for further neuroprotection studies. This is also similar to our previously published report on the effects of MPP^+^ in these cells [33].

### 2.4. BHE and Isolated Compounds Protect SH-SY5Y Cells from MPP^+^-Induced Toxicity

**Figure 6:**
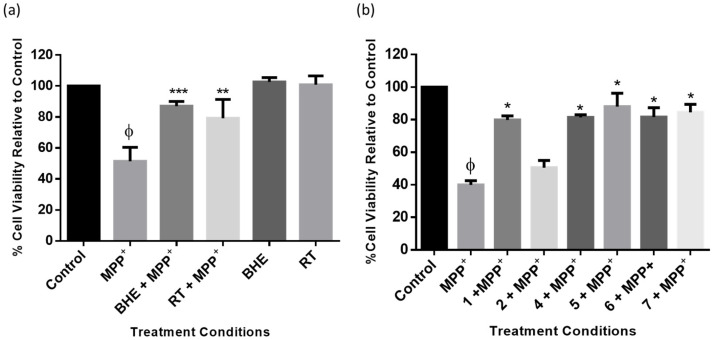
BHE and compounds show protection in SH-SY5Y cells. Cells were pre-treated with extracts (**a**) and compounds (**b**) before exposure to MPP^+^ for 24 h. Each bar represents mean percentage cell viability relative to control, and significance of difference indicated with * (*p* < 0.05), ** (*p* < 0.01), and *** (*p* < 0.001) when extract/compounds are compared to MPP^+^ and ϕ (MPP^+^ vs. control).

To investigate the neuroprotective activities of BHE and isolated compounds, SH-SY5Y cells were plated and pre-treated with 2.5 µg/mL of either BHE or RT, the standard neuroprotective agent, for 2 h before exposure to 2000 µM of MMP^+^ followed by MTT assays after 24 h. Figure 6a shows that BHE at 2.5 µg/mL significantly improved cell viability following MPP^+^ toxicity. Indeed, compared to the control, cell viability decreased to about 51% in the MPP^+^ treated group, and following pre-treatment with BHE and RT, cell viability increased to 87% and 79%, respectively. Similarly, cell viability was also improved in cells pre-treated with the compounds, and as expected, compound **2** showed no neuroprotective activity, with cell viability at approximately 50%, which discouraged further investigation (Figure 6b). Together, these results suggest that BHE and the isolated compounds could attenuate MPP^+^-induced toxicity in SH-SY5Y cells.

### 2.5. BHE Improves Cell Morphology in SH-SY5Y after MPP^+^ Insult

**Figure 7:**
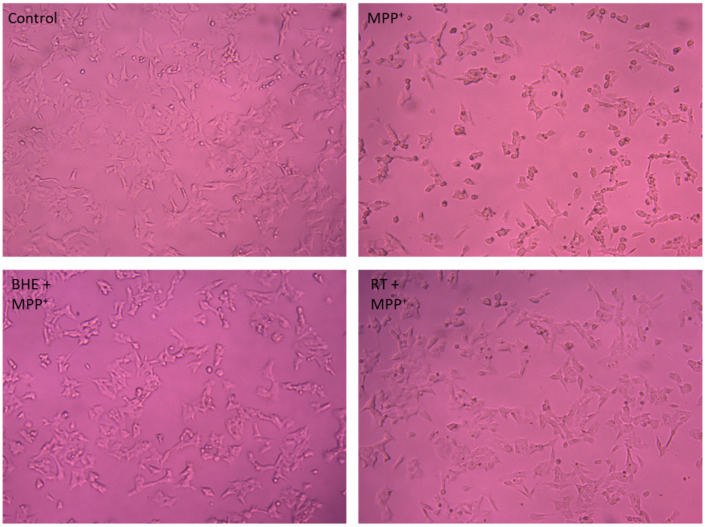
BHE and compounds inhibit SH-SY5Y morphological changes induced by MPP^+^. SH-SY5Y cells were pre-treated with BHE and compounds (2.5 µg/mL) before exposure to 2000 µM MPP^+^ for 24 h. Cells were visualized, and images acquired using the light microscope at 100× magnification.

Furthermore, morphology of the cells was observed after treatments as per the neuroprotection experiment, and Figure 7 shows that compared to the control cells, MPP^+^ treatment indeed induced loss of neuronal cells, as evidenced by the changes in cell morphology, which include loss of neuron projections and roundness of cells. However, pre-treatment of cells with 2.5 µg/mL of BHE and 25 µM RT improved cell morphology to almost that of control cells.

### 2.6. BHE and Isolated Compounds Inhibit MPP^+^-Induced Overproduction of ROS in SH-SY5Y Cells

**Figure 8:**
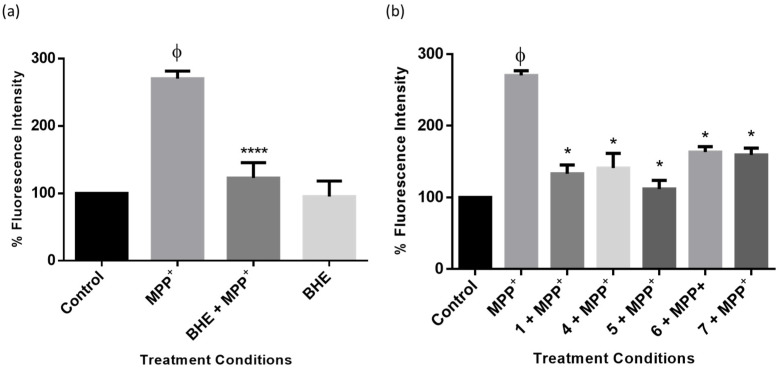
BHE and compounds inhibit MPP^+^-induced intracellular ROS production. Cells were pre-treated with 2.5 µg/mL of extracts (**a**) and compounds (**b**) before exposure to 2000 µM of MPP^+^ for 24 h, and intracellular ROS generation was determined. Each bar represents fluorescence intensity of cells expressed as percentage of control and significance of difference indicated with * (*p* < 0.05), and **** (*p* < 0.0001) when extract/compounds are compared to MPP^+^ and ϕ (MPP^+^ vs. control).

Studies have shown that impairment of ATP production as a result of a dysfunctional mitochondrial electron transport system and also the accumulation of ROS are critical steps in the cell death process in neurodegeneration following MPP^+^ toxicity [34,35]. To confirm whether the extract and compounds of *B. haemanthoides* protected SH-SY5Y cells from MPP^+^-induced ROS overproduction, cells were treated and stained with the DCFDA fluorometric dye, and fluorescence intensity was obtained. The results show that whereas MPP^+^ significantly elevated levels of ROS production compared to control cells, the cells treated with BHE and compounds had a significant reduction in cellular ROS levels (Figure 8). Together, these results show that inhibiting overproduction of ROS is in part a mechanism of protection of BHE and compounds.

### 2.7. BHE and Isolated Compounds Mitigate MPP^+^-Induced ATP Depletion in SH-SY5Y Cells

**Figure 9:**
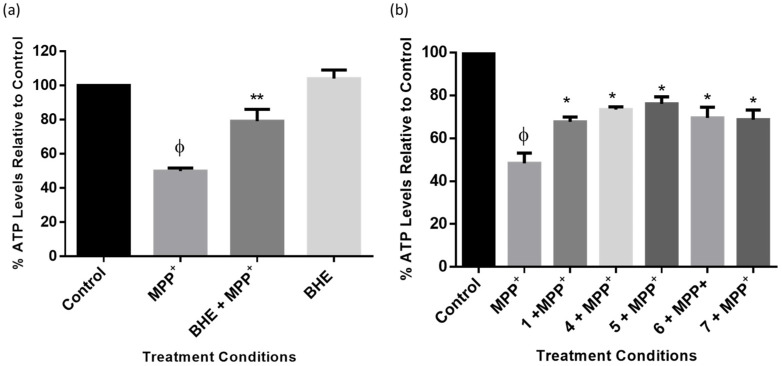
BHE and compounds inhibit MPP^+^-induced ATP degeneration. Cells were pre-treated with 2.5 µg/mL of extracts (**a**) and compounds (**b**) before exposure to 2000 µM of MPP^+^ for 24 h and ATP level assessment. Each bar represents mean percentage level relative to control, and significance of difference indicated with * (*p* < 0.05) and ** (*p* < 0.01) when extract/compounds are compared to MPP^+^ and ϕ (MPP^+^ vs. control).

As a mechanism of toxicity, MPP^+^ induces ATP degeneration in neuronal cells by the inhibition of mitochondrial complex I [36]. Thus, to further elucidate the mechanism of neuroprotection induced by BHE and isolated compounds, levels of ATP were measured in the cells after treatment, as per the neuroprotection experiment above. The results show that MPP^+^ depleted ATP levels in the cells to approximately 50%, and following pre-treatment with BHE, ATP levels increased in the cells to approximately 79% (Figure 9a). Additionally, a similar trend was observed for the cells pre-treated with compounds, and as observed with the cell viability neuroprotection results, the triterpene 5 also had the best outcome, as it increased ATP generation the most when compared to other compounds (Figure 8b). Together, these results indicate that BHE and the compounds could rescue SH-SY5Y cells from MPP^+^-induced ATP depletion.

### 2.8. BHE and Isolated Compounds Inhibit MPP^+^-Induced Apoptosis in SH-SY5Y Cells

**Figure 10:**
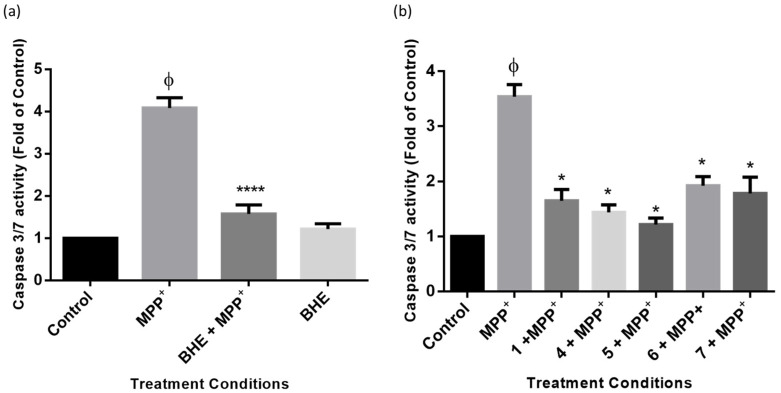
BHE and compounds reduce MPP^+^-induced caspase 3/7 activity. Cells were pre-treated with 2.5 µg/mL of extracts (**a**) and compounds (**b**) before exposure to 2000 µM of MPP^+^ for 24 h, and activity of caspase 3/7 was determined. Each bar represents level of caspase 3/7 expressed as fold of control, and significance of difference indicated with * (*p* < 0.05) and **** (*p* < 0.0001) when extract/compounds are compared to MPP^+^ and ϕ (MPP^+^ vs. control).

To further ascertain the mechanism involved in the neuroprotection of BHE and compounds in SH-SY5Y cells, the levels of cellular apoptosis were assessed using caspase 3/7 as a marker. Caspases belong to the family of cysteine proteases, which drive apoptosis in cells and carry out their function by the cleavage of substrates [37,38]. Caspases could be initiator caspases (caspases 8 and 9) or executioner caspases (caspase 3 and 7), the latter being frequently used as markers of apoptosis [39,40]. To investigate apoptosis, cells were treated as per the neuroprotection studies above, and caspase 3/7 activities were measured. Figure 10a shows that BHE mitigated MPP^+^-increased levels of caspase 3/7 activity in the SH-SY5Y cells. In particular, MPP^+^ increased levels of caspase 3/7 to about 4 times the value of the control, and pre-treatment with BHE was found to reduce this activity to about 1.5 times the control. Furthermore, all the compounds also protected SH-Y5Y cells from MPP^+^-induced apoptosis, as expected (Figure 10b). Altogether, these results indicate that the inhibition of apoptosis by BHE and the compounds is a neuroprotection mechanism.

## 3. Discussion

In the present study, we investigated the neuroprotective potentials of BHE and its bioactive compounds in an MPP^+^ model of PD. Our findings show that a total of seven compounds including three known alkaloids, two triterpenes, and tyrosol, as well as 3-hydroxy-1-(4′-hydroxyphenyl)-1-propanone were isolated from BHE (Table 2 and Figure 1). Furthermore, the neuroprotective activity of compounds **1**, **2**, **4**–**7** were determined alongside the total extract, BHE. Findings show that BHE and the compounds protected SH-SY5Y cells from MPP^+^-induced toxicity at the 2.5 µg/mL concentration. This suggests that in the presence of a neuronal insult, the extract under study, as well as the isolated compounds, could prevent loss of dopaminergic neurons in the substantia nigra pars compacta of the midbrain, which is a classical hallmark of PD. This finding is consistent with what we have previously reported in our laboratory for *Crossyne guttata*, *Nerine humilis*, and *Clivia miniata* from the same Amaryllidaceae family [41,42] and supports the traditional uses of Amrayllidaceae members for treatment of mental and neuro-related diseases [43].

Furthermore, all the isolated compounds showed neuroprotective activity, except for 1α,3α-diacetylnerbowdine (**2**), which showed toxicity to the SH-SY5Y cells even at the 2.5 µg/mL concentration. Interestingly, while most of the activities of the plants of the Amaryllidaceae family have been attributed to their alkaloids [20,21,22], and the triterpenes isolated from the BHE also showed potent neuroprotective activity comparable to the Amaryllidaceae alkaloids [44]. Specifically, the triterpene, cholest-4-en-3-one, showed the highest neuroprotective activity, which is consistent with the cell viability data (Figure 4b and Figure 6b), albeit not significant compared to other compounds. In support of our findings, previous studies have shown that pre-treatment with triterpenes protected rat primary cultures and SH-SY5Y cells against the toxicity induced by exposure to glutamate and 6-hydroxydopamine respectively [45,46]. It is interesting to indicate that the majority of alkaloids isolated so far from Amaryllidaceae have lipophilic features and avoiding polar hydroxylic/carboxylic groups in addition to the presence of nitrogen atoms, which play an important role in pH-dependent solubility. Such features could support the penetration of the BBB; however, further in vitro studies are required to understand the potential of these compounds, especially the lipophilic triterpenes isolated in this study and similar compounds.

Part of the pathogenesis of PD neuronal cells is the over-generation of ROS, as well as a reduction in the levels of ATP production [47,48]. Reported clinical studies have provided evidence that reducing oxidative stress levels could potentially lower the risk of PD. Studies have shown that MPP^+^ binds to mitochondrial complex I, which in turn, leads to the over production of ROS in the neuronal cells [34,35]. Importantly, mitochondrial complex I is critical for the maintenance of the electron transport chain, which is needed for ATP generation in the cells, thus making the mitochondria and ROS reversal promising targets for development of novel PD therapies [49,50]. Indeed administration of antioxidants such as vitamin C and E as well as coenzyme Q has shown promising outcomes as PD therapies in both animals and in patients [51,52,53,54]. In the present study, consistent with what has been previously reported for MPP^+^ [55], we showed that MPP^+^ significantly increased ROS production and this was accompanied with a depletion in ATP production (Figure 8). Furthermore, the inhibition of the elevated production of ROS by MPP^+^ and subsequently the improvement of ATP production by BHE and compounds (Figure 9) is an indication of neuroprotection. It is also well established that an increase in the levels of intracellular ATP is an indication of improved mitochondrial function, which is critical for cell survival [56,57]. A previous study has reported that *Boophone disticha*, the other member of the *Boophone* genus, protected SH-SY5Y cells from 6-hydroxydopamine-induced dopaminergic neuronal death by restoring ATP levels in the cells [26].

More so, following ROS generation, the mitochondrial cell death pathway is activated and this is also typical of the pathology of PD [58,59]. ROS accumulation, in turn, leads to mitochondrial membrane potential discharge, cytochrome c release, and the activation of caspase-3, which in all leads to cell death [55,60]. Consistent with this, MPP^+^ leads to the activation of caspase 3/7, and treatment with BHE and compounds leads to the reversal of these elevated levels of caspase activities (Figure 10). While ATP is needed for caspase activation and the depletion of ATP prevents caspase activities [61], it is plausible that reduction of ATP to about the 50% observed in this study is not enough to completely halt caspase activation. However, findings from the present study are similar to what has been previously reported for MPP^+^-induced caspase activation despite depletion of ATP [55,62]. Although not investigated, it possible that other forms of cell death like necrosis may be activated by MPP^+^ in this study, as it has been previously reported that MPP^+^ induced necrosis [63]. Altogether, BHE and compounds showed inhibition of the apoptotic pathway in the MPP^+^-treated cells, as evidenced by the reduction of caspases 3/7 activity, which is an indication of improved cell survival and cellular function.

## 4. Materials and Methods

Organic solvents such as acetonitrile (ACN, HPLC grade), methanol, dichloromethane, ethyl acetate, and hexane were purchased from Merck (Cape Town, South Africa). Normal-phase silica gel 60 PF_254_ pre-coated aluminum plat (Merck) was used for TLC analysis. Silica gel 60 H (0.040–0.063 mm particle size, Merck, Cape Town, South Africa) and Sephadex LH-20 (Sigma-Aldrich, Cape Town, South Africa) were used for column chromatography.

NMR spectra were recorded on an Avance 400 MHz NMR spectrometer (Bruker, Rheinstetten, Germany) in CDCl_3_ using the solvent signals as the internal reference. Agilent Technologies 7820A coupled with MSD5977E was used for GC–MS analysis. A 1.0 µL quantity of each sample (1.0 mg/mL CH_2_Cl_2_) was injected directly into the GC–MS operating in the electron ionization (EI) mode at 70 Ev and utilizing an HP5 MS column (30 m, 0.25 mm i.d., film thickness 0.25 m). The temperature was programmed as follows: gradient increase from 40 to 80 °C in 8 min, then 80–220 °C at 10 °C/min, hold at 220 °C for 5 min, then 220–300 °C at 20 °C/min, and 10 min hold at 300 °C. The injector and detector temperatures were kept at 250 °C, with source and MS Quad at 230 °C and 150 °C, respectively, and a 1.5 mL/min flow-rate of carrier gas (He) was maintained. A split ratio of 1:3 was applied.

### 4.1. Plant Material

*B. haemanthoides* was collected from the Northern Cape Province, South Africa, in December 2016, and sample identities were authenticated by Prof Christopher Cupido, Botany Department, University of Fort Hare. A voucher specimen (UFH 2020-3-01) of the plant was deposited in the Giffen Herbarium of the University of Fort Hare.

### 4.2. Isolation of Compounds

Fresh bulbs (~3.2 kg) were extracted at room temperature with methanol for 48 h. The total *Boophone haemanthoides* extracts (BHEs) were combined and, after evaporation, yielded ~150 g. A portion of the extract (~120 g) was chromatographed on a silica gel column (18 × 35 cm) and eluted with a gradient of hexane and ethyl acetate of increasing polarity to give 20 main fractions. The chromatographic manipulation of fractions 4, 7, 8, 10 and 12 yielded 10 known compounds in small quantities except for compounds **4**–**7** included in this study. More experimental details are contained in our recently published paper [28]. Other fractions containing alkaloids were subjected to chromatographic purification and resulted in the isolation of compounds **1**–**3** as follows: fraction 13 (1.1 g) was chromatographed on sephadex using isocratic 10% aqueous ethanol, and prep-TLC using DCM:MeOH (95:5) to yield compound **1** (40 mg). Fraction 14 was chromatographed under the same conditions to yield compound **2** (27 mg). Fraction 6 was subjected to HPLC purification using ACN:H_2_O gradient (from 50 to 100 ACN in 30 min) to yield compound **3** (~1.0 mg).

### 4.3. Physical and Spectroscopic Data of the Isolated Compounds

Distichamine (**1**): GC–MS: R_t_ 28.048 min; Mass: 329.2 (C_18_H_19_NO_5_), *m*/*z*: 398.5, 285.3, 207.1, 137.1; ${\left[\alpha \right]}_{25}^{D}N$ − 45.4 (*c* 0.1 in CH_2_Cl_2_). ^1^H-NMR (400 MHz, CDCl_3_): and ^13^C-NMR (100 MHz, CDCl_3_): see Table 2.

1α,3α-Diacetylnerbowdine (**2**): GC–MS: R_t_ 35.508 min; Mass: 403.4 (C_21_H_25_NO_7_), *m*/*z*: 344.5, 284.3, 254.2, 204.2; ${\left[\alpha \right]}_{25}^{D}N$ − 16.1 (*c* 0.1 in CH_2_Cl_2_). ^1^H and ^13^C-NMR (400/100 MHz, CDCl_3_) see Table 1.

Hippadine (**3**): GC-MS: R_t_ 34.807 min; Mass: 263.06 (C_16_H_10_NO_3_), *m*/*z*: 205.2, 177.1, 131.2; ^1^H-NMR, *δ*_H_ 8.03 (*d*, H_12_, *J* = 3.5 Hz), 7.97 (*s*, H_7_), 7.91 (1H, *d*, *J* = 7.6 Hz, H_1_), 7.74 (1H, *d*, *J* = 7.6 Hz, H_3_), 7.65 (*s*, H_10_), 7.46 (1H, *t*, *J* = 7.6 Hz, H_2_), 6.88 (1H, *d*, *J* = 3.5 Hz, H_11_), 6.15 (*s*, OCH_2_O) [29].

### 4.4. Cell Culture and Maintenance

The SH-SY5Y human neuroblastoma cells used for this study were a generous donation from the Blackburn Laboratory, University of Cape Town, South Africa. Cells were grown in Dulbecco’s modified Eagle’s medium supplemented with 10% fetal bovine serum (Gibco, Life Technologies Corporation, Paisley, UK) and 1% 100 U/mL penicillin and 100 µg/mL of streptomycin (Lonza Group Ltd., Verviers, Belgium) and maintained at 37 °C in humidified air with 5% CO_2_. Cell growth medium was changed routinely, and sub-culturing was done using of 0.25% trypsin EDTA (Lonza Group Ltd., Verviers, Belgium) when cells attained 70 to 80 percent. Passage number of cells used for this experiment were kept under 20, and cells were routinely checked for mycoplasma, and only mycoplasma free cells were used for experiments.

### 4.5. Treatments

For treatments, BHE and its compounds were dissolved in dimethyl sulfoxide (DMSO) (Sigma-Aldrich, St Louis, MO, USA) to achieve a concentration of 40 mg/mL from which further concentrations were prepared by dilution in cell growth medium. Following this, cytotoxicity screening of BHE and its compounds was performed to identify non-toxic concentrations for neuroprotection studies. Briefly 10,000 cells were seeded per well in 96-well plates and exposed to increasing concentrations (2.5, 5, and 10 µg/mL) of BHE as well as the compounds **1**, **2**, **4**, **5**, **6** and **7** (Table 2). To ensure that the amount of DMSO at the highest treatment concentration was not toxic to the cells, the control cells were treated with a similar amount of DMSO diluted in medium in the 10 µg/mL concentration. Furthermore, a quantity of MPP^+^ (Sigma-Aldrich, St Louis, MO, USA) was weighed and dissolved in un-supplemented DMEM to arrive at a concentration of 50 mM, from which the 500, 1000, 1500, 2000, and 2500 µM concentrations were prepared, and untreated cells were used as control. Treatment duration for all experiments was 24 h and the 2.5 µg/mL concentration for BHE, and its compounds and the 2000 µM MPP^+^ were chosen for further studies.

For neuroprotection studies, SH-SY5Y cells were grown in 96-well plates and pre-treated with either 2.5 µg/mL of BHE or the compounds for 2 h before adding 2000 µM MPP^+^, and treatment lasted for 24 h. Cells treated with growth medium were used as controls, and 25 µM of rutin (RT), a known neuroprotective agent, was used as a positive control.

### 4.6. Cell Viability Assays

Cells were seeded in 96-well culture plates and exposed to either BHE, compounds, MPP^+^, or with both BHE/compounds and MPP^+^ (neuroprotection experiment) for 24 h. Thereafter, cell viability was assessed using MTT (Sigma-Aldrich, St Louis, MO, USA) assays, and depending on the final volume in wells, 10 or 20 µL of 5 mg/mL MTT solution in PBS (Lonza Group Ltd., Verviers, Belgium) was added to each well and left to incubate in the dark at 37 °C for 4 h. The resultant MTT crystals formed were dissolved with DMSO, and absorbance was read at 570 nm using a microplate reader (BMG Labtech Omega^®^ POLARStar, Offenburg, Baden-Württemberg, Germany). The percentage cell viability was expressed relative to the control cells.

### 4.7. Cell Morphology

Changes in cell morphology after treatments were observed in the SH-SY5Y cells after treatment as per neuroprotection studies with BHE and RT, as stated above, and after 24 h cells were visualized using a Zeiss inverted light microscope with a 10× objective lens. Images of cells were acquired using Zeiss software Version 2.3.

### 4.8. Adenosine Triphosphate Assay

To determine the effect of treatments on mitochondrial function using ATP as an indicator, a Mitochondrial ToxGlo ATP assay kit (Promega, Madison, WI, USA) was utilized. Cells were grown in 96-well plates and treated as per neuroprotection experiments with BHE, as well as compounds, and thereafter ATP levels in treated and untreated cells were determined following the manufacturer’s procedures. Luminescence was read using a microplate reader (BMG Labtech Omega^®^ POLARStar), and the percentage levels of ATP in the treated cells were calculated relative to the control.

### 4.9. Caspase 3/7 Apoptosis Assay

To ascertain the level of apoptosis in cells following treatments, a caspase 3/7 assay kit (Promega, Madison, WI, USA) was utilized following the manufacturer’s procedures. Cells were plated in white 96-well plates at a density of 10,000 cells per well, and after treatment of cells as per neuroprotection studies with BHE and compounds, a volume of caspase 3/7 assay solution similar to what was contained in each well was added to the wells. Thereafter, luminescence was read using a microplate reader (BMG Labtech Omega^®^ POLARStar), and values obtained were expressed as fold of control.

### 4.10. Determination of Intracellular ROS

To determine changes in levels of intracellular ROS production, SH-SY5Y cells were seeded in black 96-well plates and allowed to attach overnight. Thereafter, cells were exposed to BHE and compounds prior to the addition of MPP^+^. After treatment, cells were washed with PBS and stained with 20 µM of 2′,7′-dichlorofluorescin diacetate (DCFDA, Sigma-Aldrich, St Louis, MO, USA) fluorescent dye prepared in un-supplemented for 1 h, and after which cells were washed and treated with dye containing medium replaced with PBS. Fluorescence intensity was read using a microplate reader (BMG Labtech Omega^®^ POLARStar), and values obtained were expressed as percentage of control.

### 4.11. Statistical Analysis

Data generated from this study were expressed as means ± standard error of means of at least three independent experiments analyzed using GraphPad Prism Version 6. Significance between groups was determined using one-way analysis of variance (ANOVA), and a value of *p* < 0.05 was considered as significant.

## 5. Conclusions

In this study, the neuroprotective activity of BHE and isolated compounds was investigated in an in vitro PD model using MPP^+^. Seven compounds were isolated from *B. haementhoids*, and six of the compounds were further investigated for their neuroprotective potentials. Our results show that whereas MPP^+^ induced cellular toxicity through the inhibition of cell viability, reduction in ATP levels and the induction of apoptosis, pre-treatment with BHE and the compounds attenuated these effects of MPP^+^. While these results look interesting, in future studies we hope to incorporate differentiated SH-SY5Y cells as well as investigate other mechanisms of neuroprotection, which are currently lacking in the present study. Furthermore, five of the six compounds investigated displayed varying levels of neuroprotection, except for compound **2**, which showed a reduction in cell viability and consequently no neuroprotection. As a limitation to this study, we did not ascertain whether this reduction in cell viability was a result of alteration in cell metabolism or actual cell death; thus, an experiment that involves determination of lactate dehydrogenase activity could be performed to elucidate this. Due to the wide spectrum of activities demonstrated by the Amaryllidaceae alkaloids, other metabolites, such as triterpenes, were overlooked. Surprisingly and interestingly, triterpenes and other non-alkaloidal metabolites showed strong neuroprotection activity with large safety margins when compared to alkaloids. Altogether, this study demonstrates that the Amaryllidaceae plant family may be useful in the exploration of potential neuroprotective agents, and more mechanistic and in vivo studies will be required in the future to further elucidate their activities.

## Figures and Tables

**Table 1:** ^1^H (400 MHz) and ^13^C-NMR (100 MHz) data for compounds **1** and **2** in CDCl_3._

	Compound 1		Compound 2		
	*δ*_C_	*δ*_H_ (*J*_Hz_)	*δ*_C_	*δ*_H_ (*J*_Hz_)	HMBC (H→C)
1	198.7 *s*		68.3 *d*	5.71 *br t* (2.6)	C_2_, C_10b_, C_4a_, C_3_, CO
2	102.0 *d*	5.37 *d* (1.1)	28.6 *t*	1.95 * (2α)	
				2.29 *dq* (2.6, 16.2) (2β)	C_4_, C_10b_, C_4a_, C_1_, C_3_
3	173.2 *s*		68.0 *d*	5.15 *br quint* (2.6)	C_2_, C_4_, C_4a_, C_1_, C_3_
4-α	30.3 *t*	2.49 *dd* (17.3, 6.7)	30.5 *t*	2.08 *	
4-β		2.38 *ddd* (17.3, 11.5, 1.1)		1.46 *ddd* (3.3, 12.2, 15.5)	C_10b_, C_4a_
4a	66.1 *d*	3.51 *dd* (10.8, 6.8)	59.9 *d*	3.48 *dd* (5.5, 12.2)	C_4_, C_11_, C_12_, C_6_, C_10a_
6-α	57.7 *t*	4.12 *d* (17.4)	57.5 *t*	4.13 *d* (17.3)	C_11_, C_12_, C_10_, C_6a_, C_8_, C_10a_, C_7_, C_9_
6-β		3.73 *d* (17.4)		3.76 *d* (17.3)	C_12_, C_4a_, C_6a_, C_8_, C_10a_, C_7_, C_9_
6a	116.6 *s*		117.2 *s*		
7	139.8 *s*		140.4 *s*		
8	135.3 *s*		133.2 *s*		
9	147.5 *s*		148.4 *s*		
10	100.2 *d*	7.71 *s*	97.1 *d*	6.11 *s*	C_10a_, C_6a_, C_10b_, C_7_, C_9_
10a	133.4 *s*		137.2 *s*		
10b	49.8 *s*		46.8 *s*		
11 *exo*	41.4 *t*	2.17 *ddd* (13.0, 10.4, 6.7)	38.2 *t*	1.99 *	
11 *endo*		2.30 *ddd* (12.8, 8.5, 3.6)		1.88 **	
12 *exo*	52.5 *t*	3.38 *ddd* (13.0, 10.4, 3.6)	51.0 *t*	3.30 *ddd* (13.6, 10, 3.2)	C_6_
12 *endo*		2.86 *ddd* (15.0, 13.4, 6.7)		2.76 *ddd* (13.6, 9.0, 6.3)	C_4a_, C_11_, C_6_
1-**CO**CH_3_			170.5 *s*		
1-CO**CH_3_**			21.2 *q*	1.90 ** *s*	CO(C_1_)
3-**CO**CH_3_			170.1 *s*		
3-CO**CH_3_**			21.3 *q*	1.97 * *s*	CO(C_3_)
3-OCH_3_	55.6 *q*	3.74 (*s*)			
7-OCH_3_	58.8 *q*	3.95 (*s*)	59.1 *q*	3.95 *s*	C_7_
OCH_2_O	100.3 *t*	5.84/5.85, *d*/each (1.5)	100.5 *t*	5.83/;5.79 *d*/each (1.3)	C_8_, C_9_

*, ** overlapped signals; *s* singlet; *d* doublet; *ddd* doublet of doublet of doublet; *br* broad; *quint* quintet; *t* triplet; *q* quartet.

**Table 2:** Table showing list of compounds isolated from *Boophone haemanthoides* extract (BHE).

Compound	Name
**1**	Distachamine
**2**	1α,3α-Diacetylnerbowdine
**3**	Hippadine
**4**	Stigmast-4-ene-3,6-dione
**5**	Cholest-4-en-3-one
**6**	Tyrosol
**7**	3-Hydroxy-1-(4′-hydroxyphenyl)-1-propanone
